# Visuospatial ability is associated to 2D laparoscopic simulator performance amongst surgical residents

**DOI:** 10.1016/j.sopen.2022.11.004

**Published:** 2022-12-07

**Authors:** Hanne Pedersen, Darya Ståhl, Mikael Ekelund, Magnus Anderberg, Martin Bäckström, Anders Bergenfelz, Kristine Hagelsteen

**Affiliations:** a Practicum Clinical Skills Centre, Skåne University Hospital, Lund, Sweden; b Lund University, Skåne University Hospital, Department of Clinical Sciences, Surgery, Lund, Sweden; c Lund University, Skåne University Hospital, Department of Clinical Sciences, Surgery, Malmö, Sweden; d Lund University, Skåne University Hospital, Department of Clinical Sciences, Paediatrics, Lund, Sweden; e Department of Psychology, Lund University, Sweden

**Keywords:** Resident selection, Resident performance, Recruitment, Selection, Visuospatial ability

## Abstract

**Background:**

The technical skills of a surgeon influence surgical outcome. Testing technical aptitude at point of recruitment of surgical residents is only conducted in a few countries. This study investigated the impact of visuospatial ability (VSA), background factors, and manual dexterity on performance in two different laparoscopic surgical simulators amongst applicants and 1st year surgical residents.

**Method:**

Applicants from general surgery, pediatric surgery, and urology were included from seven hospitals in Sweden between 2017 and 2021. Some 73 applicants were invited and 50 completed. Participants filled out a background form, and were tested for manual dexterity, and visuospatial ability. Two laparoscopic simulators were used, one 2D video box trainer and one 3D Virtual Reality Simulator.

**Results:**

A significant association was found between the visuospatial ability test and 2D video box laparoscopic performance (95 % CI: 1.028–1.2, p-value <0.01). For every point on the visuospatial test the odds of accomplishing the task increased by 11 %. No association was found between VSA and performance in a laparoscopic VR simulator using 3D vision. No other background factors were associated with performance in the two laparoscopic simulators.

**Conclusion:**

Visuospatial ability in applicants to surgical residency positions is associated to performance in a 2D video box trainer. Knowledge of a resident's visuospatial ability can be used to tailor individualized laparoscopic training programs, and in the future might function as a selection tool concerning laparoscopic ability.

**Key message:**

Visuospatial ability differs greatly amongst applicants for surgical residency and is associated to laparoscopic simulator performance. Testing applicants' visuospatial ability could possibly be used to tailor individualized laparoscopic training programs or in the future as a selection tool concerning laparoscopic ability.

## Highlights

• The visuospatial ability test is a low cost and feasible assessment tool.
• The visuospatial ability correlates to performance in a 2D video box trainer.
• Background factors did not correlate to performance in laparoscopic simulators.

## Graphical abstract

**Figure:**
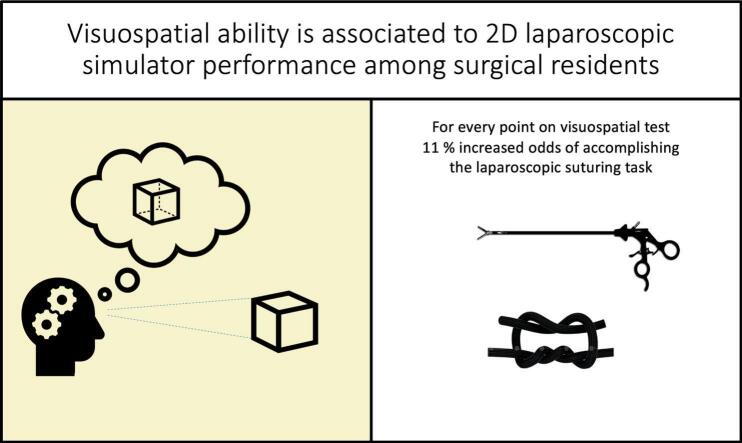

## Background

Technical skills of surgeons influence surgical outcome. Testing of technical abilities has been discussed as a part of surgical resident selection for several years [[1], [2], [3], [4], [5]]. However only a few countries use testing in a structured manner [6]. With the implementation of laparoscopic surgery and thereby new technical demands, studies indicate that some struggle to reach proficiency [3,[7], [8], [9]]. Although still debated, laparoscopic simulator performance seems to be transferrable to skills in the operating room [[10], [11], [12], [13]]. Choosing the right surgical applicant is therefore a matter not only of time and money spent on the selection process and subsequent training, but also of importance for patient safety [4,14,15]. A valid tool to identify individuals with limited technical aptitude hence seems to be of importance.

Visuospatial ability (VSA), the ability to mentally visualize, rotate and transform objects in three-dimensional space, and manual dexterity have been discussed as a possible proxy variable for laparoscopic ability and technical skill. In a recent review on the subject, which included 75 articles, a positive association was seen between surgical performance and VSA [16]. Further, results from the Purdue Pegboard have been correlated with novice endovascular skill [17]. Several background factors such as sex and gaming experience have also been suggested to be predictive of aptitude [18].

In a decentralized selection system for surgical residents, for example in Sweden [6], a standardized method for selection of candidates to surgical training positions needs to be affordable, and easy to implement and assess. A previous study has revealed that there is a lack of transparency of assessments in the current Swedish selection process. This may lead to the recruitment of unsuitable candidates [19].

The primary aim of this study is to investigate the impact of demographic factors, manual dexterity and VSA on the performance in two different laparoscopic surgical simulators amongst applicants to surgical resident positions and 1st year residents. A secondary aim is to examine whether there was a correlation of performance of the residents between the two simulators.

## Method

### Participants and setting

The study included all applicants called to interviews for a locum position or surgical training position in general surgery, urology, and pediatric surgery in seven hospitals in the Southern Swedish Health Care Region and Uppsala University hospital between 2017 and 2021. All departments agreed to take part in the study and forwarded contact information when hiring or interviewing. Informed written consent was obtained from all participants. Participants were queried regarding background variables, such as sex, age, self-assessed 3D vision, dominant hand, gaming, education, year of certification, previous work experience, surgical experience for open and laparoscopic procedures, current employment status and hospital, experience of simulator training.

The participants underwent tests chosen to estimate visuospatial ability (VSA), dexterity and laparoscopic skill in two different simulators. Background forms were collected and managed using REDCap electronic data capture hosted by Lund University, Sweden [20,21].

Ethical approval was obtained from the Swedish Ethical Review Agency (approval number 2016/1050). This study is part of a longitudinal study where surgical residents are followed throughout their surgical training until provided with a specialist diploma (Certificate of Completion of Specialist Training, CCST).

### VZ-3 Surface Development test

**Fig. 1:**
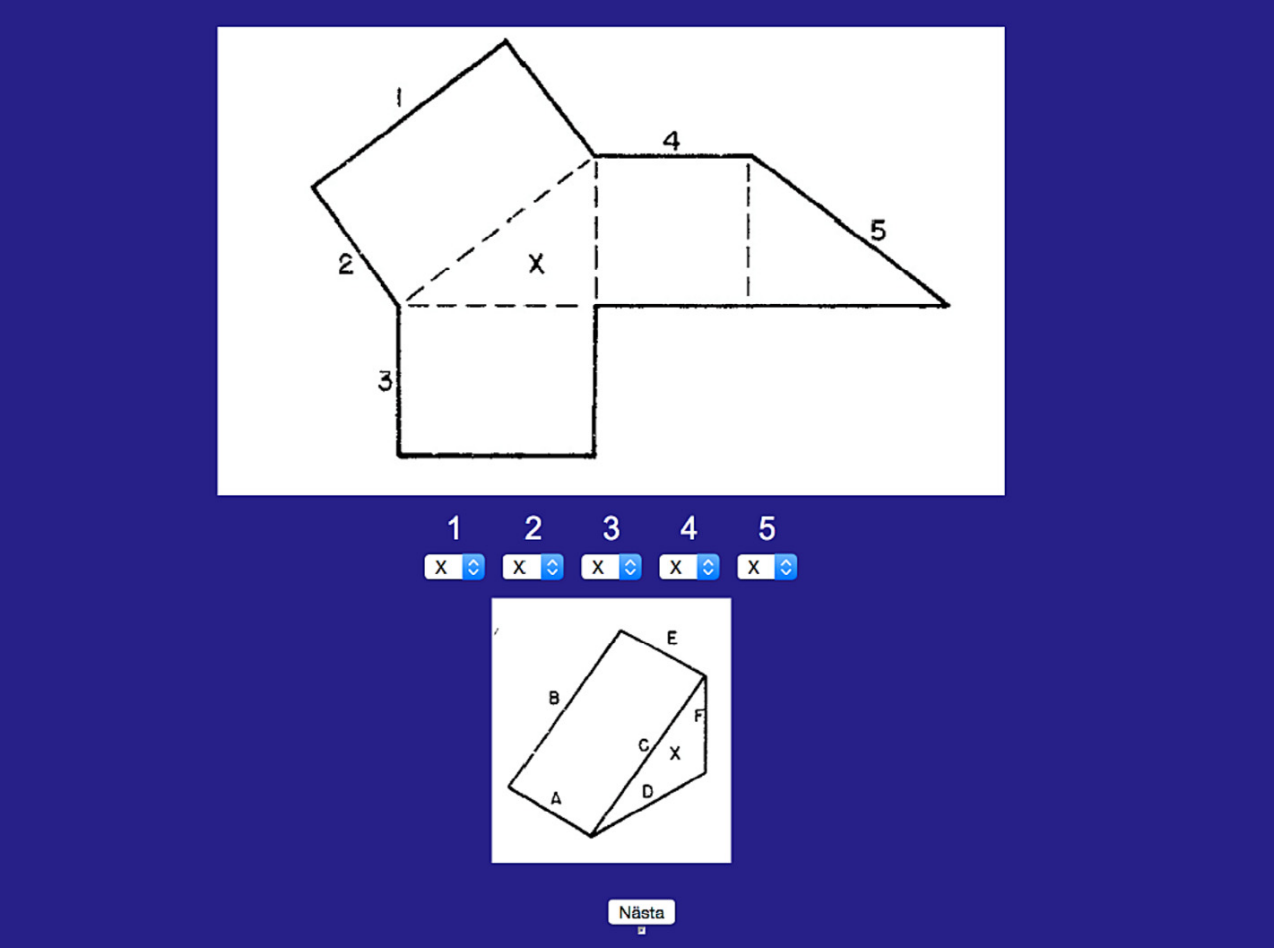
One of 12 tasks from VZ-3 Surface Development test, requesting participants to visualize how a flat shape can be folded to form a predefined object.

VZ-3 Surface Development test (Kit of Factor-References Cognitive Tests) [22] is a cognitive test used for measuring spatial ability, requesting participants to visualize how a flat shape can be folded to form a predefined object (Fig. 1). An individually coded link to a computer-based version of VZ-3 Surface Development test was sent to all participants to execute at home. The test consisted of 12 different tasks and maximum time to complete each task was set to 1 min. The maximum test score was 60 points.

### Purdue Pegboard

The Purdue Pegboard (PP) is used to test finger and hand dexterity. Instructions were obtained from the manufacturer [23] and translated into Swedish. All participants conducted three rounds of tests and the median was calculated for the sum (right hand + left hand + both hands) and assembly. The median score of assembly was chosen as measure for dexterity due to its increased complexity.

### Surgical simulators, performance score and video assessment

LapSim® (Surgical Science, Gothenburg, Sweden) is a laparoscopic virtual reality (VR) simulator that is equipped with a three-dimensional display monitor and a haptic hardware platform for proprioceptive sensation force feedback [24]. The Ahlberg exam [10], which consists of six tasks needed to be passed twice to succeed, was conducted. This test will be repeated during the longitudinal study trial and anticipated to be very difficult at this point of testing due to inexperienced participants. A change in the original order of tasks was therefore done to making “lift & grasp” the first task, and only the result from this task was included in the study. The “lift & grasp” task time limit is 43 s. There were no restrictions on the number of attempts. The total time for the simulator test was 20 min and included three standardized warming up tasks to get acquainted with the simulator and settings. Individual feedback and instructions from an experienced surgeon were given throughout the test. The total score and pass/fail of the task were used as outcome parameters.

Simball® Box (Surgical Science, Gothenburg, Sweden) is a 2D hybrid video box trainer that record a video and performance metrics using authentic laparoscopic instruments [25,26]. A suture pad with a simulated incision, a 12 cm 3-0 braided suture on a SH needle (Vicryl®, Ethicon) and two 5 mm authentic needle holders (Olympus) were used. An instruction video demonstrating a double overhand knot was shown for the participant, followed by 5 min of practice with further instructions from an experienced laparoscopic surgeon. The instruction video was shown once more, and an attempt without instructions was recorded by video. Time limit for the exercise was set to five-minutes. Metrics were obtained through the laser pattern in the Simball Box trocars, measuring distance travelled of all instruments in 4 dimensions; horizontal, vertical, in-out and rotation [25]. Although these are objective metrics, the quality of the knot is still best measured visually on a recorded video. A short, measured distance or time does not automatically correspond to a correct quality of the three throws in the surgeon's knot. The video recordings were analyzed independently by three experienced surgeons according to a previously validated score [27], with modifications according to the given exercise and instructions (Appendix A). The outcome was set to *pass/fail*. Agreement amongst the assessors was required, and disagreements were solved by renewed analyses by the group. Since the outcome was set to pass/fail, no interrater reliability was calculated.

### Statistical analysis

Binary outcomes were modeled using logistic regression, and continuous outcomes using linear regression. Both univariable and multivariable analyses were performed. When the number of independent variables was too many, compared to the number of observations, only variables with p-values below 0.3 was included in the multivariable models. For logistic regression models, odds ratios were presented along with 95 % confidence intervals (CI) and for linear regression beta coefficients with 95 % confidence intervals were presented. The association between Lapsim® and Simball® Box was analyzed using odds ratio. All analyses were performed in cooperation with a professional statistician using IBM SPSS Statistics for Windows, Version 25.0.

## Results

Some 73 applicants were invited to participate, 53 (73 %) accepted and 50 individuals completed the study. The most common reasons for not participating were non-responding (n = 10) or lack of time/schedule/geographical location issues (n = 7). Only a few of those not included in the study were offered a locum or surgical training position.

Background variables are shown in Table 1. The median age was 30 (range 27–41) years and 30 (60 %) were men. All, except one of the participants had experience from assisting in laparoscopic procedures, with a range from “never” to “>100 times”. For independent laparoscopic operations the experience ranged from “never” [34] to “51–80 times” [1].

**Table 1:** Background variables for participants in the study.

		Count
Sex	Female	20
	Male	30
Age, median and range (years)	30 (27–41)	
Years after medical license, median and range	2 (0–16)	
Self-assessment: “I can see 3D on movies”	No	0
	Yes	50
Gaming experience	No	25
	Female	15
	Male	10
	Yes	25
	Female	5
	Male	20
Dominant hand	Right	44
	Left	4
	Both	2
Work status	Trainee	34
	Locum	9
	No employment offered	7
Laparoscopic simulator training experience	No	24
	Women	14
	Men	10
	Yes	26
	Women	6
	Men	20
Assisted in laparoscopic procedures	Never	1
	1–10 times	14
	11–20 times	15
	21–50 times	14
	51–80 times	1
	81–100 times	2
	>100 times	3
Independent laparoscopic experience	Never	34
	1–10 times	11
	11–20 times	1
	21–50 times	3
	51–80 times	1
	81–100 times	0
	>100 times	0

All participants completed the Purdue Pegboard and Lapsim® tests. Three values from VZ-3 Surface Development test and one videorecording from Simball® Box were missing due to technical failures of the equipment. For 20 of the 49 video recordings from Simball® Box there was an initial disagreement between the three assessors on pass/fail before reaching consensus.

Results from statistical analyses are found in Table 2, Table 3, Table 4.

**Table 2:** Regression model between pass/fail results from Simball® Box, included background factors, and tests. No association between performance in the laparoscopic simulator was identified related to background factors like sex, age, gaming and surgical experience. Variables with p-values below 0.3 are included in the multivariable analysis.

	OR (95 % CI)	p-Value
*Univariable analysis*		
Sex (male vs female)	2.50 (0.67, 9.39)	0.175
Simulator experience	0.94 (0.29, 3.11)	0.921
Independent laparoscopic experience	0.91 (0.25, 3.27)	0.884
Gaming experience	2.26 (0.66, 7.70)	0.192
Visuospatial ability	1.11 (1.03, 1.20)	0.008⁎
Pegboard “assembly”	1.03 (0.93, 1.13)	0.627

*Multivariable analysis*		
Sex	3.13 (0.55, 17.97)	0.200
Gaming experience	0.93 (0.17, 5.00)	0.929
Visuospatial ability	1.11 (1.03, 1.21)	0.010⁎

⁎ p < 0.05.

**Table 3:** Regression model between VZ-3 Surface Development test, sex, and gaming experience.

	Beta (95 % CI)	p-Value
*Univariable analysis*		
Sex	0.70 (−6.64, 8.04)	0.848
Gaming experience	4.20 (−2.90, 11.30)	0.239

*Multivariable analysis*		
Sex	−1.60 (−9.83, 6.62)	0.696
Gaming experience	4.93 (−3.15, 13.00)	0.225

**Table 4:** Regression models for binary and continuous outcome on LapSim® and included background factors and tests. In univariable analysis for the continuous outcome on Lapsim® associations were found for sex and gaming experience. This association disappeared in the multivariable analysis. Variables with p-values below 0.3 are included in the multivariable analysis.

	OR (95 % CI)	p-Value
*Binary outcome*		
Univariable analysis		
Sex (male vs female)	1.71 (0.45, 6.58)	0.432
Simulator experience	1.11 (0.31, 3.92)	0.877
Independent laparoscopic experience	1.48 (0.39, 5.54)	0.563
Gaming experience	1.88 (0.52, 6.85)	0.337
Visuospatial ability	1.00 (0.95, 1.06)	0.897

*Continuous outcome*		
Univariable analysis		
Sex	9.68 (0.04, 19.31)	0.049⁎
Simulator experience	3.80 (−5.98, 13.58)	0.439
Independent laparoscopic experience	−0.49 (−11.03, 10.06)	0.927
Gaming experience	9.62 (0.19, 19.05)	0.046⁎
Visuospatial ability	0.30 (−0.13, 0.72)	0.166
Multivariable analysis		
Sex	8.25 (−3.09, 19.59)	0.150
Gaming experience	4.64 (−6.66, 15.94)	0.412
Visuospatial ability	0.25 (−0.17, 0.67)	0.229

⁎ p < 0.05.

In the laparoscopic simulator tests, 13 participants passed “*lift and grasp*” on LapSim® (3D VR simulator), 16 passed tying a knot in Simball® Box (2D box trainer) and five participants passed both. Neither of the three participants that had assisted in laparoscopic surgery >100 times passed the simulator tasks in both LapSim® and Simball® Box. The odds ratio for passing the test in LapSim® for those participants that had already passed the test in Simball® Box were 1.42 (0.38, 5.34), but the result was not significant.

A significant association was found between the VZ-3 Surface Development test and Simball® Box (95 % CI: 1.028–1.2, p-value <0.01), which remained after multivariable analysis (Table 2). With every point on the VZ-3 Surface Development test the odds of accomplishing the task increased by 11 %. There was no significant association with gaming experience, manual dexterity results from the Purdue Pegboard or other background factors (Table 2). No significant association between sex and gaming experience on the result from VZ-3 Surface Development test was identified (Table 3).

In univariate analysis for the continuous outcome on LapSim® weak associations were found for sex and gaming experience [28]. The associations disappeared in the multivariable analysis (Table 4). No association was found between the VZ-3 Surface Development test and the 3D VR environment in LapSim®.

## Discussion

This study demonstrates associations between the performance on laparoscopic knot tying in Simball® Box, a 2D video box trainer, and visuo-spatial ability (VSA), measured by VZ-3 Surface Development test. No associations between performance in the two laparoscopic simulators were identified related to background factors like sex, age, gaming and surgical experience. In this study, individuals passing the laparoscopy test in the 2D simulator test had 1.42 times higher odds of passing the virtual reality 3D simulator test, although not significant.

A prediction of the learning potential and educational needs during residency could be informative for the employer who seeks to secure the return of educational and monetary investment in a new employee. Previous studies have indicated that 8–15 % struggle to progress or to reach technical proficiency in laparoscopy and thus represent slow learners or low performers [3,7,9]. Around one third of the participants passed either of the laparoscopic simulator tests, and five passed both, but it is too early to know whether this result is applicable to performance during the entire residency, or if the exercise might have been too hard. Being part of a longitudinal study, aiming to follow residents throughout residency, follow-up testing will hopefully clarify this. To use laparoscopic simulators or other tests to predict future laparoscopic performance at the point of selection of surgical residents is appealing since technical skill proficiency is associated with fewer complications and thus a matter of patient safety [4]. Even though this study focuses on technical skill, this is only one part of being a competent surgeon, and non-technical skills are equally important for patient safety [19,29].

The present study showed a significant association between VSA and performance in a 2D box trainer, with every point on the VZ-3 Surface Development test increasing the odds of success by 11 %. The lack of association with test outcome in the 3D laparoscopic simulator (LapSim®) could be explained by the 3D setting automatically present visuospatial depth. Participants with limited VSA may therefore be able to compensate for this by using 3D vision. Conflicting results have been shown for VSA as a predictor of future performance [16,18]. It has been hypothesized that VSA is a trait that is fixed in adulthood, with no differences between novices and experienced surgeons and therefore seemingly not trainable [30]. On the contrary, others have found improved results with training [31,32], but to date few studies have been conducted [32]. A recent review suggested that videogaming did not increase the VSA [33]. A multitude of different VSA tests have been used in previous studies, which could possibly explain conflicting results to date [32]. If VSA indeed is untrainable, and easy to test, it would be promising to use as a selection method. However, this assumes the effect is not only found at baseline but consistent over time. On the other hand, our result could also suggest the possibility of adjusting for VSA deficit by 3D laparoscopic technic in the operating room and simulated environment.

The results pose the question if the set of visuospatial skills differ depending on surgical modality. The 2D video box trainer is similar to the setup used in standard laparoscopic surgery, and hence the difference in results between 2D and 3D simulators are interesting. Other studies comparing 3D simulators to 2D simulators have shown shortening of learning curves and improved results for box trainers and VR simulator with 3D [[34], [35], [36]]. No major difference has been shown between performance in 2D box trainers and 2D VR trainers amongst residents, apart from a small possible benefit of using real instruments in the box trainer [37].

To fully evaluate VSA as a predictor of future resident performance, learning curves and time to independent perform laparoscopic procedures should be investigated. Further knowledge of the development of VSA is also needed. If poor VSA of the resident leads to prolonged time to reach proficiency, or even lack of proficiency, the VSA test could be useful to implement during the selection process for surgical residents. Moreover, it will enable departments to identify residents with the need for additional tutoring and coaching in laparoscopic surgery.

Several factors, such as sex and previous gaming experience, have been suggested to relate to laparoscopic performance. However, there is no consensus to date on which factors are most relevant [18,38]. A large review found videogaming promising as indicator for baseline simulator performance, further highlighting the problem of stratification and differences in measures in the included studies [38]. Prior videogaming experience as a yes/no question could have a huge range in hours. A continuous scale is probably preferred to fully elaborate on the effect. In this study, the use of a binary variable as well as a rather small sample could be one reason for not reaching significance.

This study did not find manual dexterity to be predictive of laparoscopic performance, though previous studies have shown promising results [17,39,40]. Other background factors have also been suggested having an impact on laparoscopic performance, such as sex, playing musical instrument, and previous laparoscopic training [18,33]. Louridas et al. found previous laparoscopic experience to be the only background factor predictive of baseline laparoscopic skill [18]. This is something that was not replicated in the present study. Since a dichotomous variable (yes/no) was used despite a rather large range in numbers of operations this could have impacted on the result. The numbers in each group were considered too small to be of statistic value analyze on group-level.

### Strengths and limitations

The strengths of this study are that it includes several teaching hospitals, and the applicant cohort is representative for Sweden's current recruitment system. The long inclusion time-period made the sample size larger which would otherwise have been impossible. Another strength is that the study used two different simulators thus enabling analysis of differences in predictive factors for the simulators.

The video was reviewed first individually and then together by three assessors, which increase credibility for the knot assessment. The discussion amongst the assessors, with a possibility to show the other assessors on the video how to judge a certain movement or a correct throw, decrease bias and increase objectivity.

There are several limitations in this study. Compared to Anglo-Saxon studies with centralized admission systems where every applicant is tested by default, this study includes a rather small number of participants. The selection bias of those who declined participation is difficult to predict, and this group consisted of both applicants that was accepted as locums or directly into a program, as well as rejected applicants. A large part of those who declined where rejected candidates that for example had difficulties to accept due to need for long distance travelling or living abroad, or not being able to take a day off since their current employer did not partake in the study. Of those who were included, all but one came from the same health care region in Sweden and all the region hospitals surgical and/or urological departments are represented. The current pandemic of covid-19 has further impacted on data collection by making scheduling of participants difficult. The study was further affected by changes in heads of departments in all but one department. Another limitation is the lack of objective testing of participants 3D cinema vision, using only self-reported results where all declared having 3D vision. A limitation in the instruction for rating of videos was also identified and additional instructions as well as test-videos could have been used to make the surgeons more comfortable with the form.

## Conclusion

Careful considerations should be taken before selection of candidates to surgical training positions. This study indicates that visuospatial ability could serve as a predictive factor for initial 2D laparoscopic performance. Knowledge of a resident's visuospatial ability can be used to tailor individualized laparoscopic training programs and might be used as selection tool in the future concerning laparoscopic ability.

## Funding sources

This work was funded by Prostatacancerförbundet, Löf (Löf regionernas ömsesidiga försäkringsbolag) and 10.13039/501100009780Region Skåne.

## Ethics approval

Ethical approval was obtained from the Swedish Ethical Review Agency (approval number 2016/1050).

## CRediT authorship contribution statement

**Hanne Pedersen:** Formal analysis, Investigation, Writing – original draft. **Darya Ståhl:** Formal analysis, Investigation, Writing – original draft. **Mikael Ekelund:** Methodology, Investigation, Writing – review & editing. **Magnus Anderberg:** Methodology, Investigation, Writing – review & editing. **Martin Bäckström:** Methodology, Software, Writing – review & editing. **Anders Bergenfelz:** Conceptualization, Methodology, Investigation, Writing – review & editing, Supervision. **Kristine Hagelsteen:** Conceptualization, Methodology, Investigation, Writing – review & editing, Supervision.

## Conflict of interest

None.

## Figures and Tables

**Table:** 

			Yes	No
Needle position	1	Held at 1/2 to 2/3 from the tip		
Needle driving through tissue	2	Uses tissue or other instrument for stability		
	3	Driving with one movement		
Incision exit	4	Removing the needle along its curve		
Technique of knots	5	Correct C loop (no S or O loops)		
	6	Smoothly executed throw, no fumbles		
	7	Correct inverse C loop (no S or O loops)		
	8	Smoothly executed throw, no fumbles		
	9	Knot squared (capsized reef/surgical)		
	10	Correct third C loop (no S or O loops)		
	11	Smoothly executed throw, no fumbles		
Knot quality	12	Squared and secured		
	13	Not too tight or too loose		
	14	Instruments always visible^a^		
SCORE (max 14)				

Correct knot = Yes for 5, 7, 10, 12, 12, 14, and within the time-limit of 5 min.

a Added as additional point to the original list.
